# Supplementary material for: Palbociclib Promotes Dephosphorylation of NPM/B23 at Threonine 199 and Inhibits Endometrial Cancer Cell Growth
Source: Cancers (Basel). 2019 Jul 20;11(7):1025. doi: 10.3390/cancers11071025 (PMC6678831; doi:10.3390/cancers11071025)
Supplement: Supplementary file 1 [file cancers-11-01025-s001.pdf]

Supplementary Materials: Palbociclib Promotes Dephosphorylation of NPM/B23 at Threonine 199 and Inhibits Endometrial Cancer Cell Growth

Chiao-Yun Lin, Li-Yu Lee, Tzu-Hao Wang, Cheng-Lung Hsu, Chia-Lung Tsai, Angel Chao and Chyong-Huey Lai

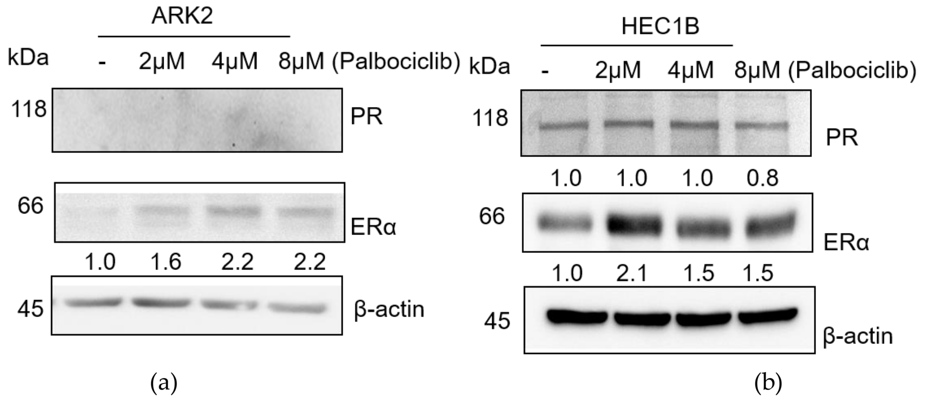

Figure S1. PR expression did not affect by palbociclib treatment. (**a**,**b**) ARK2 (left panel) and HEC1B cells (right panel) were treated with vehicle (−) or different doses of Palbociclib (2, 4, 8μM) for 24 h. Cell lysates were subsequently resolved on SDS-PAGE and subjected to immunoblotting with antibodies raised against PR, ERα and β-actin. Densitometry-derived values (bottom) are normalized with the control that was set as 1. Data shown are derived from three independent experiments. β-actin serves as the loading control for normalization.

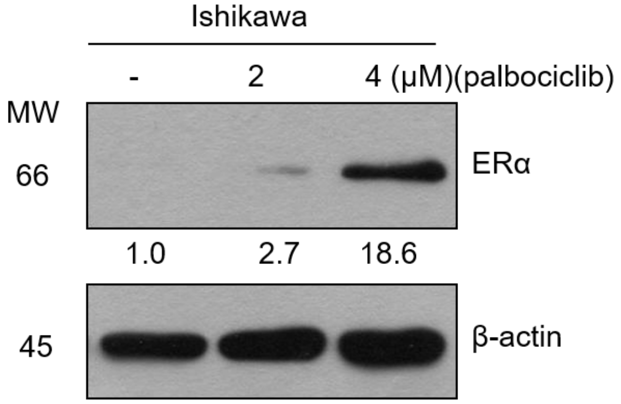

Figure S2. Palbociclib induces ERα expression in late-passage (n >40) Ishikawa endometrial cancer cells. Ishikawa were treated with vehicle (−) or different doses of Palbociclib (2, 4 μM) for 24 h. Cell lysates were subsequently resolved on SDS-PAGE and subjected to immunoblotting with antibodies raised against ERα and β-actin. Densitometry-derived values (bottom) are normalized with the control that was set as 1. Data shown are derived from three independent experiments. β-actin serves as the loading control for normalization.

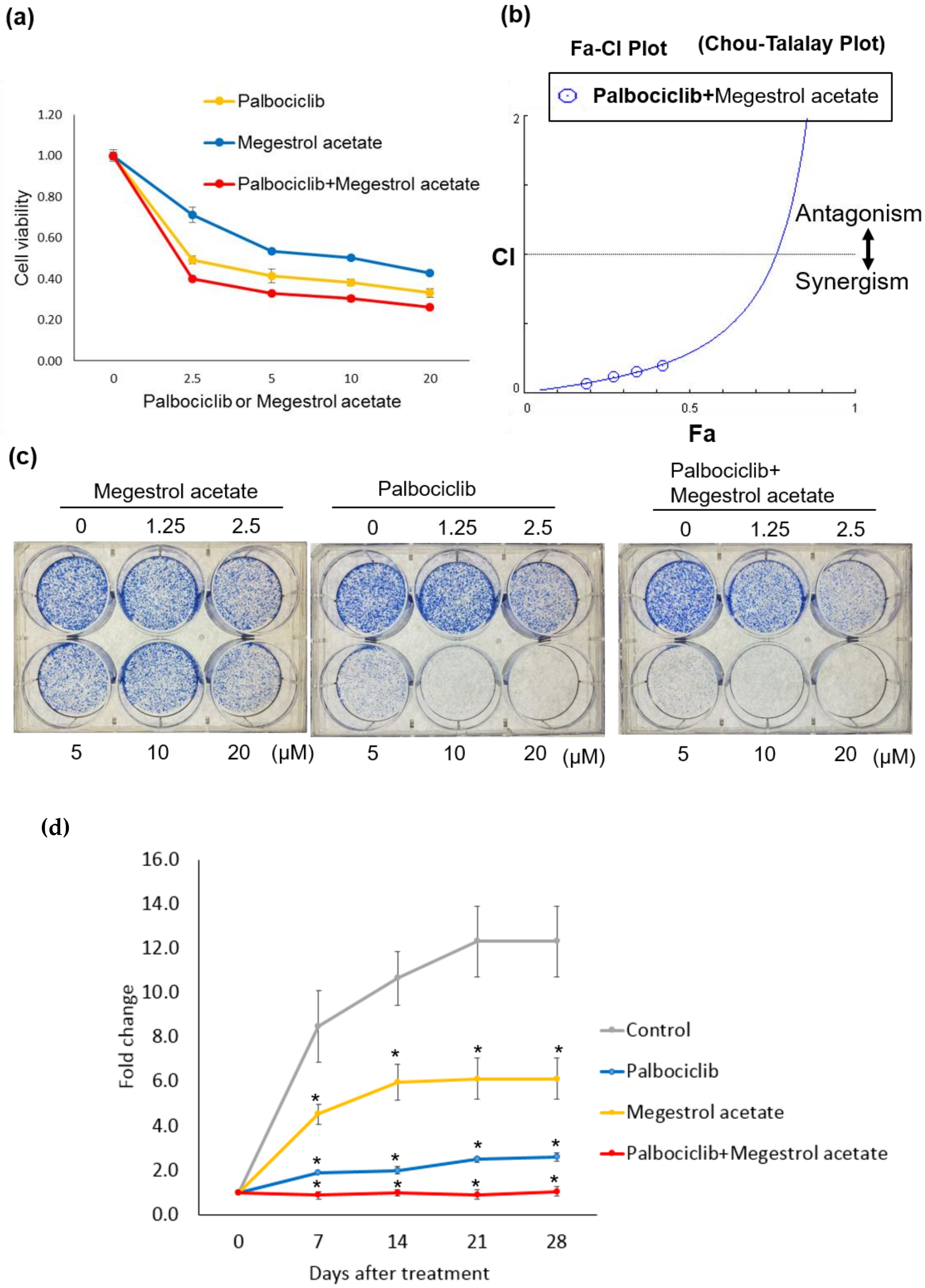

Figure S3. The combination of palbociclib and megestrol acetate exerts antitumor effects in endometrial cancer cells and in a xenograft tumor model. (**a**) HEC1B cells were treated with vehicle (-) or different doses of palbociclib alone (0, 2.5, 5, 10, and 20 μM), megestrol acetate alone (0, 2.5, 5, 10, and 20 μM), or their combination for 72 h. Cell survival was analyzed with the MTT assay. All experiments were performed in triplicate and data are expressed as fold change ± SD relative to vehicle-treated cells (left panel). (**b**) The synergistic effect of palbociclib and megestrol acetate was analyzed using the CompuSyn software (right panel). (**c**) HEC1B cells were treated with vehicle (-) or different doses of palbociclib alone (0, 1.25, 2.5, 5, 10, and 20 μM), megestrol acetate alone (0, 1.25, 2.5, 5, 10, and 20 μM), or their combination (palbociclib plus megestrol acetate) for 5 days. The clonogenic assay was used to assess colony formation. (**d**) Inhibitory effects of the palbociclib/megestrol acetate combination on cancer growth in a xenograft tumor model. * $p < 0.05$ *versus* control.

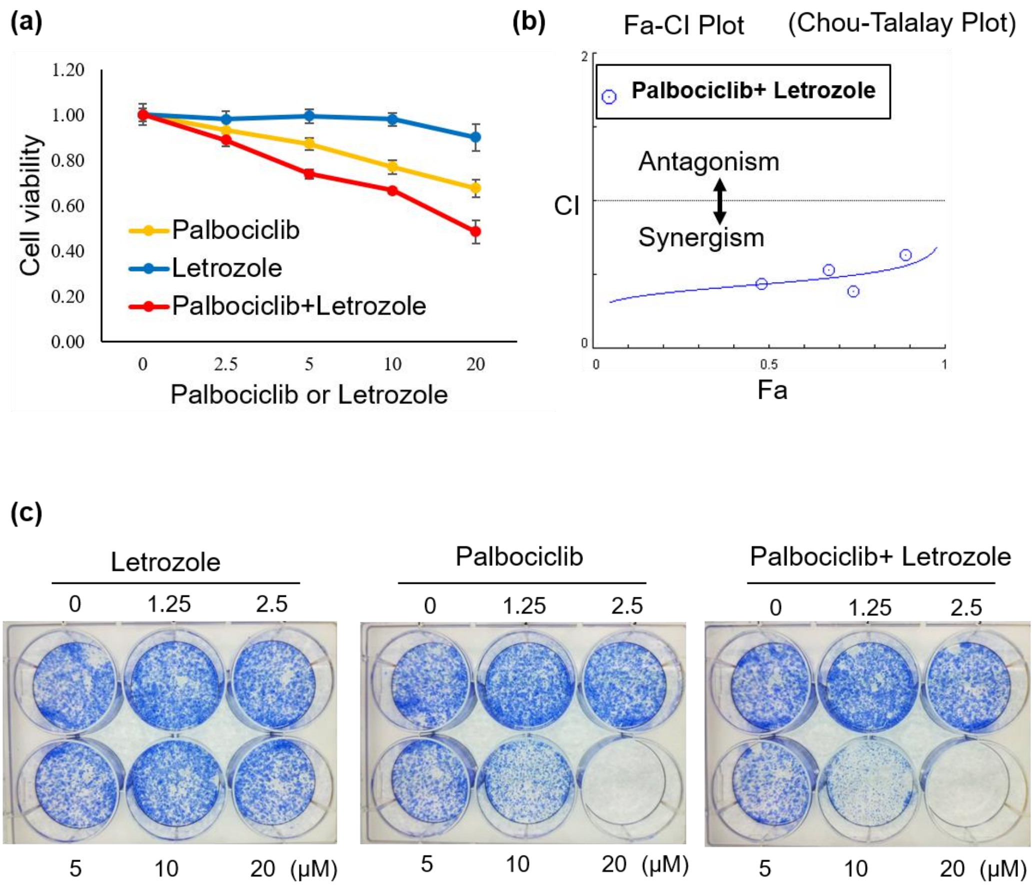

Figure S4. The combination of palbociclib and letrozole exerts synergistic antitumor effects in endometrial cancer cells. (**a**) ARK2 cells were treated with vehicle (-) or different doses of palbociclib alone (0, 2.5, 5, 10 and 20 μM), letrozole alone (0, 2.5, 5, 10, and 20 μM), or their combination for 72 h. Cell survival was analyzed with the MTT assay. All experiments were performed in triplicate and data are expressed as fold change ± SD relative to vehicle-treated cells (left panel). (**b**) The synergistic effect of palbociclib and letrozole was analyzed using the CompuSyn software (right panel). (**c**) ARK2 cells were treated with vehicle (-) or different doses of palbociclib alone (0, 1.25, 2.5, 5, 10, and 20 μM), letrozole alone (0, 1.25, 2.5, 5, 10, and 20 μM), or their combination (palbociclib plus letrozole) for 5 days. The clonogenic assay was used to assess colony formation.

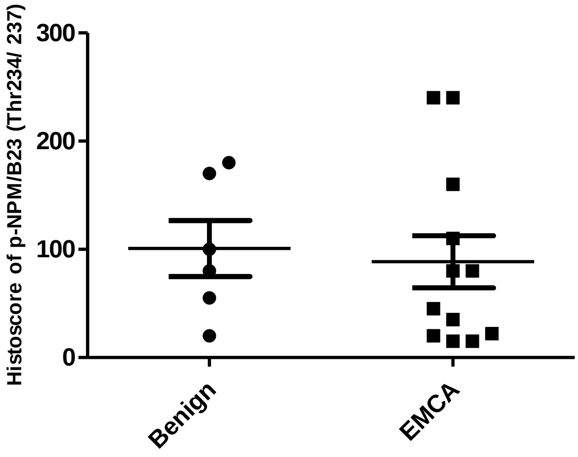

Figure S5. Immunohistochemical expression of phospho-NPM/B23 (Thr234/237) in normal endometrium and endometrial cancer. Immunohistochemistry was used to calculate histoscores of phospho-NPM/B23 (Thr199) expression in endometrial tissue obtained from patients who underwent hysterectomy for benign gynecologic conditions ($n = 6$) and endometrial cancer ($n = 12$).

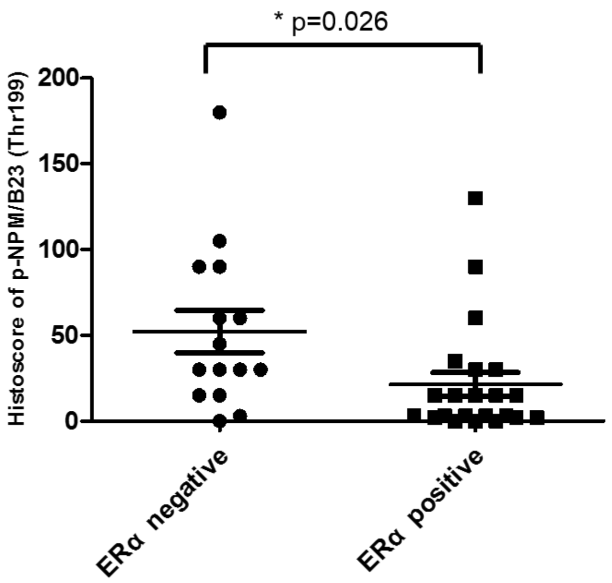

Figure S6. Immunohistochemical expression of phospho-NPM/B23 (Thr199) in endometrial cancer. Histoscores of phospho-NPM/B23 (Thr199) expression in ERα negative (n = 15) and ERα positive (n = 22) patients with endometrial cancer.

Table S1. Clinical information of patients with endometrial cancer

Number	Age	Type
1	52	endometrioid adenocarcinoma
2	58	adenosquamous carcinoma
3	51	endometrioid adenocarcinoma
4	54	endometrioid adenocarcinoma
5	49	endometrioid adenocarcinoma
6	56	adenosquamous carcinoma
7	72	endometrioid adenocarcinoma
8	61	endometrioid adenocarcinoma
9	46	endometrioid adenocarcinoma
10	44	endometrioid adenocarcinoma
11	53	endometrioid adenocarcinoma
12	59	endometrioid adenocarcinoma
13	76	clear cell adenocarcinoma
14	94	clear cell adenocarcinoma
15	67	serous papillary cystadenocarcinoma
16	53	clear cell adenocarcinoma
17	62	serous papillary cystadenocarcinoma
18	50	serous papillary cystadenocarcinoma
19	78	clear cell adenocarcinoma
20	62	serous papillary cystadenocarcinoma
21	65	serous cystadenocarcinoma
22	56	serous adenocarcinoma
23	84	serous adenocarcinoma

24	43	clear cell adenocarcinoma
25	59	serous adenocarcinoma
26	72	serous adenocarcinoma
27	72	serous adenocarcinoma
28	65	clear cell adenocarcinoma
29	62	serous adenocarcinoma
30	46	clear cell adenocarcinoma
31	56	clear cell adenocarcinoma
32	70	serous adenocarcinoma
33	68	clear cell adenocarcinoma
34	64	serous adenocarcinoma
35	79	serous adenocarcinoma
36	76	clear cell adenocarcinoma
37	73	clear cell adenocarcinoma

© 2019 by the authors. Licensee MDPI, Basel, Switzerland. This article is an open access article distributed under the terms and conditions of the Creative Commons Attribution (CC BY) license (http://creativecommons.org/licenses/by/4.0/).
